# Efficient Detection of Proteins Retro-Translocated from the ER to the Cytosol by *In Vivo* Biotinylation

**DOI:** 10.1371/journal.pone.0023712

**Published:** 2011-08-24

**Authors:** Gianluca Petris, Lara Vecchi, Marco Bestagno, Oscar R. Burrone

**Affiliations:** International Centre for Genetic Engineering and Biotechnology, Trieste, Italy; Ohio State University, United States of America

## Abstract

Retro-translocation from the ER to the cytosol of proteins within the secretory pathway takes place on misfolded molecules that are targeted for degradation by the cytosolically located 26S proteasome complex. Retro-translocation occurs also for other proteins (such as calreticulin) that, despite being synthesized and transported to the ER, are in part dislocated to the cytosol. We have taken advantage of the *E. coli* derived biotin-ligase (BirA) expressed in the cytosol of mammalian cells to specifically biotin-label *in vivo* proteins within the secretory pathway that undergo retro-translocation. We validated the method using four different proteins that are known to undergo retro-translocation upon different conditions: the human trans-membrane protein MHC class-I α chain (MHC-Iα), the Null Hong Kong mutant of the secretory α1 anti-trypsin (NHK-α1AT), the immunoglobulin heavy chain (HC) and the ER chaperone calreticulin (Crt). We observed specific mono-biotinylation of cytosolically dislocated molecules, resulting in a novel, reliable way of determining the extent of retro-translocation.

## Introduction

A critical common feature of the biogenesis of proteins that enter the secretory pathway, either to be secreted or membrane-anchored, is transport across the ER membrane to reach the lumen or to be inserted into membranes [1]. Once in the ER, newly synthesized proteins initiate a complex process of folding, assisted by several ER resident chaperones belonging to different families, such as the heat shock (Hsp90/Grp94, Hsp70 and Hsp40 [2]) and lectin (calnexin, calreticulin [3]) families. In addition, several covalent post-translational modifications take place within the ER, that allow the protein to acquire the proper conformation; these modifications include disulphide bond formation, N-glycosylation and glycosylphosphatidylinositol (GPI) addition [4]. N-glycosylation is particularly important because it allows the interaction with molecular chaperones that assist glycoprotein folding, increasing protein solubility and avoiding the formation of protein aggregates. When acquisition of the correct folding fails, misfolded molecules become substrates of the quality control cellular mechanism known as ER-associated degradation (ERAD [5]).

Molecules targeted to ERAD, upon recognition by ER lectins, such as OS-9 and XTP3-B, and molecular chaperones, such as Hsp70 (BiP) or GRP94 [6], [7], are retro-translocated to the cytosol, a process also known as dislocation, for degradation by the 26S proteasome complex [8]. Retro-translocation operates also for proteins, such as calreticulin, that despite being synthesized and transported to the lumen of the ER, entail other functions in the nuclear/cytosolic compartments [9].

The mechanism of retro-translocation itself, as well as the composition of the channel involved during transport is poorly understood. Sec61, the main component of the translocon for translocation into the ER of newly synthesized proteins [10], has been associated to the putative retro-translocon channel as well [11]. However, other proteins like Der1p and Hrd1p in yeast and the family of Derlin proteins in mammals have also been described as candidate constituents of the channel [12], [13], [14]. TRAM1, previously characterized for its function during translocation of nascent polypeptides in the ER [15], has recently been involved in the disposal of misfolded membrane proteins, but not of soluble ERAD substrates [16]. In addition BAP31, a three membrane spanning ER protein, previously demonstrated to be involved in protein sorting [17], has been found to interact with the Sec61 translocon and described to be implicated in retro-translocation [18].

Detection of retro-translocated molecules relies usually on the separation of the cytosolic fraction and identification of de-glycosylated proteins accumulated in the cytosol upon proteasome inhibition [19], [20], although a tightly coupling of degradation to dislocation may hamper their detection. A limitation of this technique could be represented by a low efficiency in the detection of retro-translocated substrates, because of incomplete proteasome inhibition or the inability to identify possible glycosylated, yet retro-translocated molecules. In an attempt to improve the detection of retro-translocated substrates, we developed a method of specific *in vivo* labeling of retro-translocated proteins, by selective biotinylation in the cytosolic compartment.

Biotinylation of proteins *in vivo* can be achieved by co-expression of the *E. coli* derived biotin-ligase BirA [21] and the protein of interest tagged with a 15 aa long biotin-acceptor-peptide (BAP): this peptide contains a single lysine which is efficiently biotinylated by BirA [22]. We have previously shown that, because of its cytosolic localization, BirA needs to be engineered with a signal leader peptide, in order to translocate to the ER and to achieve efficient biotinylation of proteins within the secretory pathway [23].

Here we show, instead, that co-expressing cytosolic BirA (cyt-BirA) with defined BAP-tagged membrane bound or secretory proteins, results in biotin-labeling only of molecules dislocated to the cytosol, thus representing a simple, reliable and quantitative way of determining the extent of retro-translocation for proteins that have entered ERAD or proteins that need to dislocate to reach other cellular compartments.

## Results

**Figure 1:**
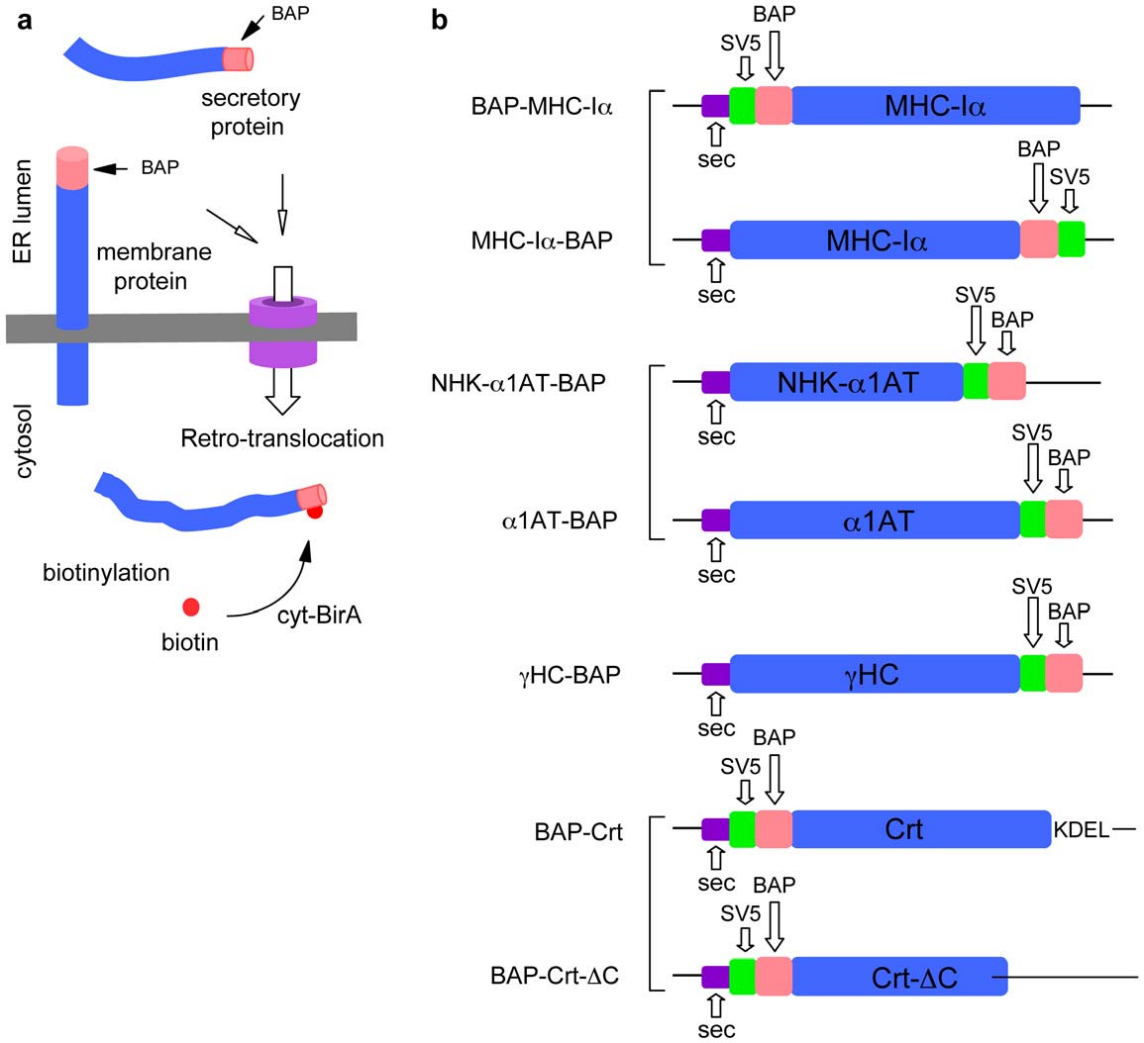
Scheme of *in vivo* biotinylation of retro-translocated proteins and of genetic constructs. (a), membrane or secretory proteins with the BAP-tag localized to the luminal side of the ER. Upon retro-translocation the cytosolic BirA covalently adds a biotin to the single acceptor lysine within BAP. (b), scheme of the principal constructs used. The BAP and SV5 tags are indicated. In each case the signal leader peptide (sec) is indicated.

Fusion of the 15 aa long BAP tag (GLNDIFEAQKIEWHE) to a protein makes possible its mono-biotinylation when co-expressed in the same cellular compartment with the *E. coli* BirA. If the protein of interest is BAP-tagged in the region located to the luminal side of the ER, it will not be biotinylated by a cytosolically localized BirA (cyt-BirA), unless it is retro-translocated to the cytosol. Therefore, the addition of the BAP tag allows the rapid biotinylation of misfolded membrane-bound or secretory proteins that enter the ERAD pathway for proteasomal degradation, as well as of ER synthesized proteins that have functions also in the cytosol or nucleus after retro-translocation to the cytosol, such as calreticulin (Fig. 1a).

In order to test the system we used four different model molecules: the A2 allele of the human major histocompatibility complex class-I α chain (MHC-Iα), the Null Hong Kong (NHK) mutant of the secretory α1 anti-trypsin (NHK-α1AT), the Ig heavy chain (HC) and the ER chaperone calreticulin (Crt). The MHC-Iα is a type-I trans-membrane protein and a well-known substrate of ERAD that undergoes retro-translocation when co-expressed with either of the two cytomegalovirus immune evasion proteins US2 or US11 [19], [24]; the NHK-α1AT is a natural truncated version of the wild type protein (α1AT) that is not secreted and is also substrate of ERAD [25], while the Ig HC, when expressed in the absence of the Ig light chain (LC), is not secreted because of its association with the ER resident chaperone grp78/BiP [26]. Crt instead, is an ER chaperone and the major Ca^2+^-binding protein within the ER lumen that, following retro-translocation, has regulatory functions both in the cytosolic and nuclear compartments [27], [9].

A schematic diagram of all the genetic constructs used is shown in Fig. 1b. The type-I transmembrane MHC-Iα was tagged with the BAP peptide at the N-terminus (BAP-MHC-Iα), therefore localizing the tag to the lumen of the ER, so that it cannot be biotinylated by cyt-BirA unless it gets retro-translocated to the cytosol. As a positive control, a C-terminus BAP-tagged version (MHC-Iα-BAP) with the BAP exposed to the cytosolic side was also constructed. Similarly, Crt was BAP-tagged at the N-terminus to avoid disturbing the C-terminal ER-retention signal KDEL. Instead, the NHK-α1AT as well as wild type α1AT (α1AT) and HC were tagged at the C-terminus. In all cases the 12 aa long SV5 tag was also included to favor recognition.

Biotinylation was monitored either by ELISA or by a Western blotting retardation assay (WB-ra) where samples, following denaturation, are run in the presence of streptavidin (StrAv). In this latter assay the complex formed by StrAv and biotinylated molecules is resistant to the SDS-PAGE denaturing conditions and therefore migration is retarded in relation to the non-biotinylated ones that do not bind StrAv.

### Retro-translocation of MHC-Iα

**Figure 2:**
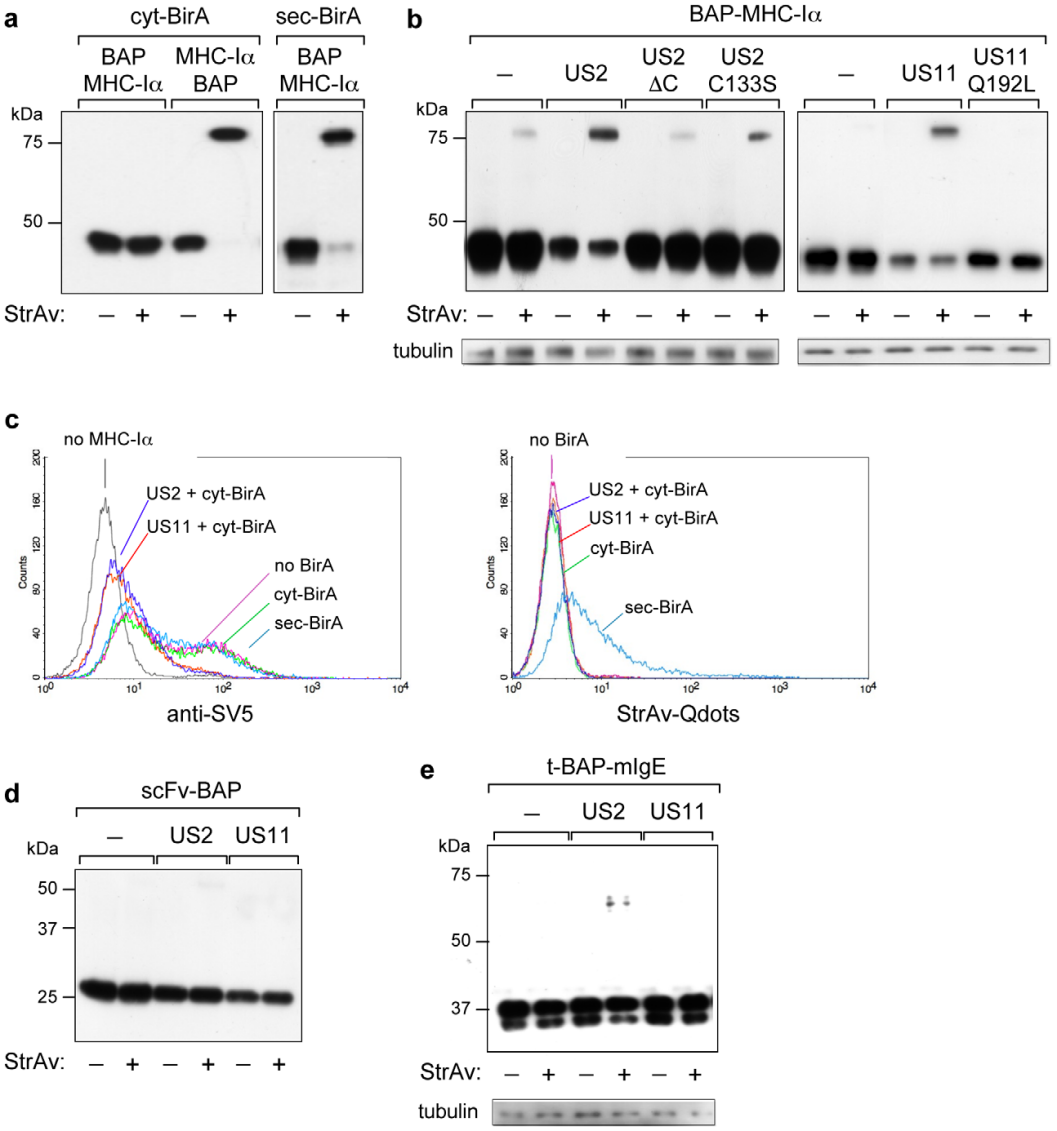
Retro-translocation of MHC-Iα. WB-ra of cellular extracts of HEK293 cells transfected with: (a) BAP-MHC-I**α** and MHC-Iα-BAP, co-expressed with cyt-BirA, or BAP-MHC-Iα co-expressed with sec-BirA, as indicated; (b) BAP-MHC-Iα and cyt-BirA and US2 or the US2 mutants US2-ΔC and US2-C133S (left panel), or US11 or US11 mutant US11-Q192L (right panel), as indicated. (c) Cytofluorimetry of HEK293 cells co-transfected with BAP-MHC-Iα and either cyt-BirA (alone or with US2 and US11), or sec-BirA, and stained with anti-SV5 mAb (left panel) or Streptavidin-QuantumDot (right panel). (d) WB-ra of supernatants of HEK293 cells co-transfected with a secretory BAP-tagged scFv control protein and cyt-BirA, BAP-MHC-Iα and US2 or US11. (e) WB-ra of cellular extracts of HEK293 cells co-transfected with t-BAP-mIgE, cyt-BirA and US2 or US11, as indicated. All blots were developed with anti-SV5 mAb, and anti-tubulin where indicated.

Fig. 2 shows an analysis by WB-ra of total cellular extracts from HEK293 cells co-transfected with cyt-BirA and either the N- or C-terminus BAP tagged MHC-Iα. As expected, while BAP-MHC-Iα was essentially not biotinylated, C-tagged MHC-Iα-BAP yielded complete biotinylation (Fig. 2a). Likewise, BAP-MHC-Iα was almost completely biotinylated by a previously described engineered version of BirA containing an N-terminal leader signal peptide (sec-BirA) that localizes to the ER [23] (Fig. 2a). When BAP-MHC-Iα was co-expressed with cyt-BirA and either of the two immunoevasins US2 and US11 to induce retro-translocation and degradation, a significant increase in the amount of biotinylated BAP-MHC-Iα (as compared to co-expression with an irrelevant protein) was observed, despite a reduction in the total amount of BAP-MHC-Iα (Fig. 2b), suggesting that molecules biotinylated by cyt-BirA corresponded to the retro-translocated ones. This was confirmed by co-expressing BAP-MHC-Iα with three different retro-translocation incompetent mutants, namely, US11-Q192L [12], US2-C133S [28] and US2-ΔC (lacking the cytosolic domain aa 186–199 [29]), which did not affect MHC-Iα expression and showed much lower biotinylation levels than wild type immunoevasins (Fig. 2b). This is therefore a clear indication that the biotinylated material actually represents molecules exposed to the cytosol that become substrate of cyt-BirA. This conclusion was further supported by cytofluorimetric analysis of the mature, cell-surface exposed MHC-Iα co-expressed with cyt-BirA (and US2 or US11) or sec-BirA (Fig. 2c). Levels of MHC-Iα on the cell membrane were reduced, as expected, when co-expressed with either US2 or US11, but were not affected by co-expression of cyt-BirA or sec-BirA, as revealed by detection with anti-SV5 antibody (Fig. 2c, left panel). When the same set of transfected cells was instead analysed with streptavidin-QuantumDot to exclusively detect biotin-labeled MHC-Iα, positive staining was observed only when co-expressed with sec-BirA and not with cyt-BirA (Fig. 2c, right panel). Thus, in cells expressing cyt-BirA all the MHC-Iα exposed on the cell surface (regardless whether expressed alone or with US2 or US11) was not biotinylated, further demonstrating the intracellular localisation of the biotinylated molecules. Furthermore, when a negative control secretory BAP-bearing protein (scFv-BAP [23]), which does not interact with US2 or US11, was co-expressed with BAP-MHC-Iα, cyt-BirA and US2 or US11 the secreted material was found not biotinylated, although somehow reduced with US11 (Fig. 2d), thus confirming the specificity of biotinylation in the cytosolic compartment. Similarly, a truncated version of human membrane IgE, irrelevant to US2 or US11, that is otherwise fully biotinylated by sec-BirA [23], was also essentially not biotinylated by cyt-BirA (Fig. 2e). The small amount of biotinylated molecules found with US2 was likely due to the immunoevasin induced cellular stress.

These results demonstrated that: i) the BAP tag is fully sensitive to biotinylation when localized to the same cellular compartment as the BirA enzyme, and ii) molecules within the ER lumen, either membrane bound or secretory, are protected from the biotinylating activity of cytosolic BirA. A small fraction of biotinylated BAP-MHC-Iα, when expressed either alone or with an irrelevant protein was frequently detected, most likely representing the fraction of misfolded molecules that have spontaneously entered the ERAD pathway. This was not due to post-lysis biotinylation since cellular extracts were prepared by directly resuspending cells in hot, SDS-containing lysis buffer to immediately denature proteins and block the activity of cyt-BirA.

Results similar to the ones shown above with WB-ra were also obtained by performing [^35^S]-Methionine pulse-chase labeling experiments, followed by analysis of immunoprecipitated MHC-Iα in a PAGE retardation assay. As shown in Fig. S1, while in cells expressing US2 30% of the [^35^S]-Methionine-labeled MHC-Iα was biotinylated after the 30 min pulse labeling period, the proportion of biotinylated molecules increased to around 55% after the 2 h chase, despite a decrease in total MHC-Iα.

### Proteasome inhibition increases biotinylation of MHC-Iα

**Figure 3:**
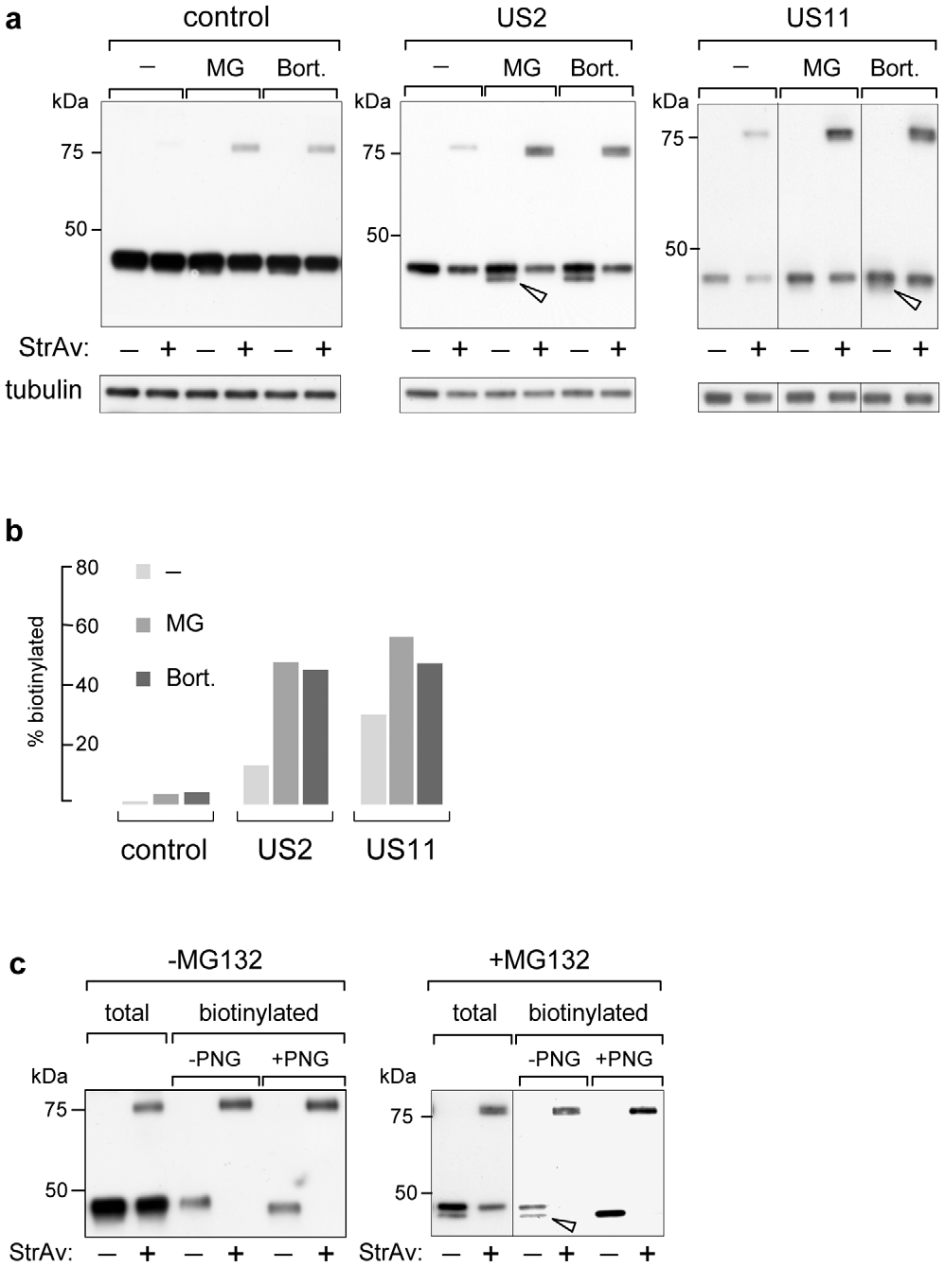
Proteasome inhibition effect on biotinylation of MHC-Iα. (a) WB-ra of cellular extracts of HEK293 cells co-transfected with BAP-MHC-Iα and cyt-BirA (control) and, where indicated, with US2 or US11 in the absence (−) or presence of MG132 (MG; 50 µM for 4 h) or Bortezomib (Bort.; 50 µM for 4 h). (b) Quantification of the relative levels of biotinylated MHC-Iα shown in (a) calculated as the ratio between biotinylated vs. non-biotinylated form in a given lane. (c) WB-ra of PNGaseF (PNG) treated, affinity-purified biotinylated BAP-MHC-Iα, derived from MG132-treated and untreated cells co-expressing US2. All blots were developed with anti-SV5 mAb and where indicated with anti-tubulin. Open arrowheads indicate de-glycosylated BAP-MHC-Iα.

The results shown above indicate that biotinylated MHC-Iα corresponds to the fraction dislocated to the cytosol and not yet degraded by the proteasome. To further support this interpretation we tested the effect of proteasome inhibitors MG132 (50 µM for 4 h) and Bortezomib (50 µM for 4 h). As shown in Fig. 3 upon proteasome inhibition an increased amount of around 5–6 fold of biotinylated molecules was detected, both in the absence and presence of US2 and US11 (Fig. 3a). In addition, although the total amount of BAP-MHC-Iα was reduced when either US2 or US11 were present, the proportion of biotinylated molecules accumulated upon proteasome inhibition was much increased. Quantification of the relative intensities between retarded (biotinylated) and non-retarded bands of Fig. 3a (displayed in Fig. 3b) showed in fact that, while less than 1% of BAP-MHC-Iα expressed alone was found biotinylated in non-treated cells, upon proteasome inhibition this proportion increased to 3–4% with MG132 or Bortezomib; when co-expressed with US2 or US11 the proportion of biotinylation raised from 13% (US2)–27% (US11) up to 43–52% following proteasome inhibition (Fig. 3b).

For MHC-Iα, it has been shown that, after dislocation in U373 cells, de-glycosylation by the cytosolically localized cellular PNGase takes place just before engagement by the proteasome, although de-glycosylation appears not to be essential for retro-translocation [30]. In HEK293 cells de-glycosylated MHC-Iα was less apparent, particularly with US11. In the presence of proteasome inhibitors (Fig. 3a), a band corresponding to de-glycosylated material was more evident and found to be, as expected, completely biotinylated. However, within the glycosylated fraction, a consistent amount was also biotinylated. Indeed, Fig. 3c shows the composition of biotinylated MHC-Iα, after affinity-purification with StrAv-coated beads from extracts of cells co-expressing US2, incubated with or without MG132. The presence of de-glycosylated material, in addition to a relevant amount of glycosylated one was clearly seen from MG132-treated samples (right panel), while in the absence of MG132, MHC-Iα was mostly glycosylated. *In vitro* PNGaseF treatment in both cases was used to confirm the state of glycosylation of the purified material. Similar results were obtained for US11. This demonstrates that a significant number of molecules already retro-translocated to the cytosol and not yet de-glycosylated (also evident in Figure S1) have already become biotinylated. These results also indicate that in these cells retro-translocation occurs more rapidly than de-glycosylation, which can therefore be considered the rate-limiting step.

### Trypsin sensitivity of biotinylated MHC-Iα

**Figure 4:**
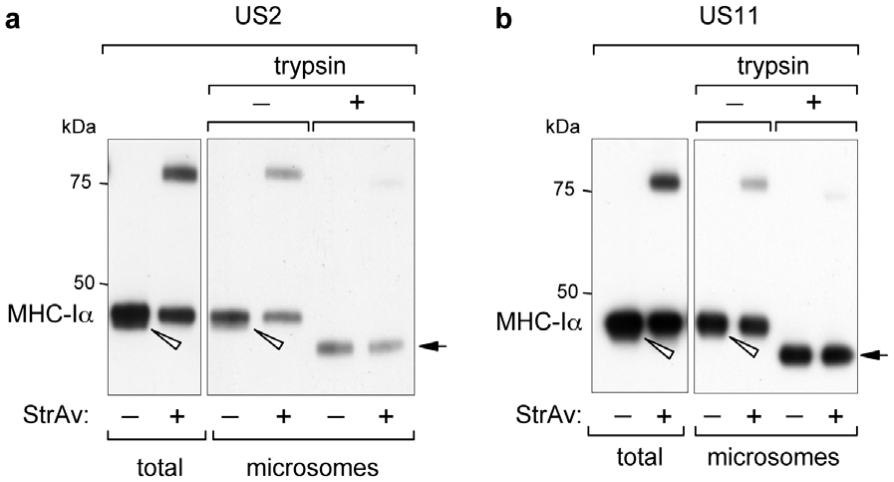
Trypsin sensitivity of biotinylated MHC-Iα. WB-ra of microsomes-containing cell lysates (microsomes) derived from cells expressing BAP-MHC-Iα, cyt-BirA and US2 or US11 and treated or not with trypsin, as indicated. As a control, an aliquot of cells was directly lysed in SDS sample buffer (total). Before lysis cells were treated with 10 µM MG132 for 16 h. Open arrowheads indicate de-glycosylated molecules, while arrows indicate MHC-Iα with the cytosolic C-terminal tail digested by trypsin.

To further demonstrate the specificity of biotinylation occurring only on molecules that have been exposed to the cytosolic side, trypsin-sensitivity experiments were performed on microsomes-containing cell lysates. Cells co-expressing MHC-Iα, cyt-BirA and US2 or US11 were gently lysed in an appropriate buffer to preserve the ER structure and then treated with trypsin. As shown in Fig. 4, the non-biotinylated material obtained with both US2 (Fig. 4a) or US11 (Fig. 4b) was trypsin-resistant (except for the removal of the cytosolic C-terminal tail, that results in a faster migrating band), as expected for molecules protected because of their localisation in the luminal side of the ER. The biotinylated material, however, was degraded by trypsin, thus demonstrating that the biotinylated MHC-Iα molecules are exposed to the cytosolic side.

### Retro-translocation of secretory proteins

**Figure 5:**
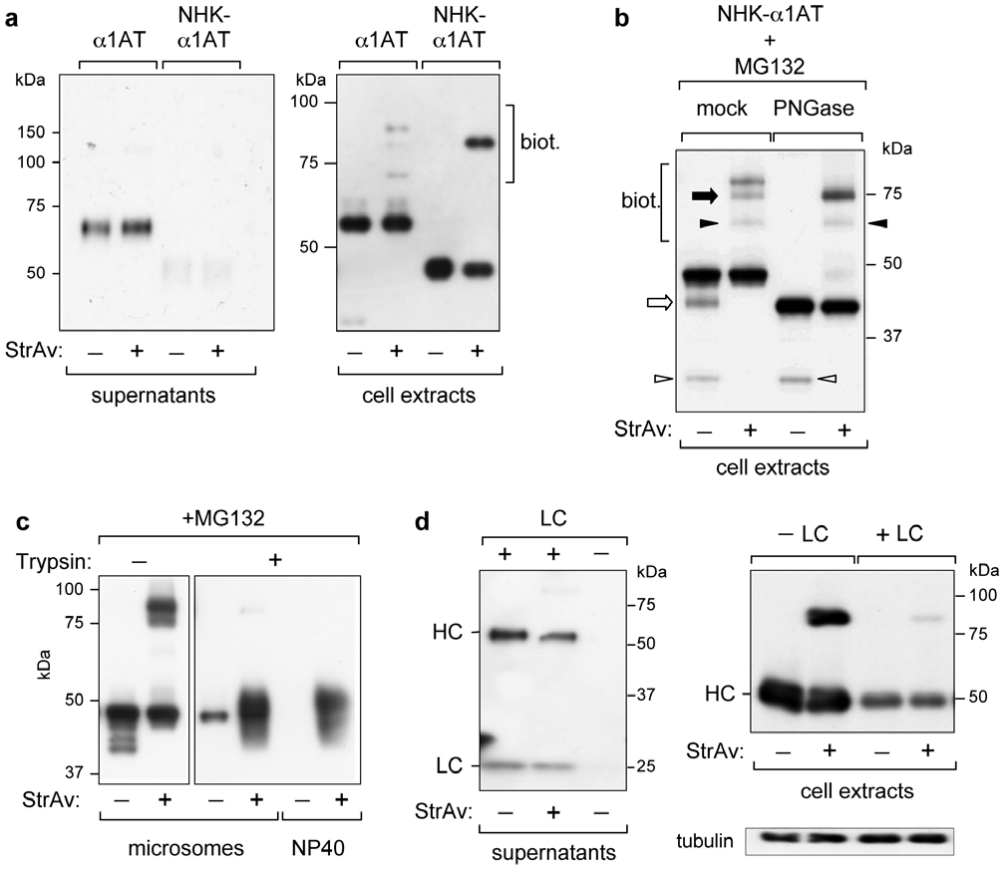
Retro-translocation of secretory proteins. WB-ra of supernatants and/or cellular extracts, as indicated, of HEK293 cells co-transfected with: (a), α1AT-BAP or the mutant NHK-α1AT-BAP and cyt-BirA; (b), NHK-α1AT-BAP and cyt-BirA in the presence of MG132 (10 µM for 16 h) and digested or not (mock) with PNGaseF. Open arrow and arrowheads indicate de-glycosylated full-length NHK-α1AT-BAP and NHK-α1AT-BAP fragments, respectively, while filled arrow and arrowheads indicate the corresponding biotinylated bands. (c) WB-ra of microsomes-containing cell lysates (microsomes) derived from cells expressing NHK-α1AT-BAP and cyt-BirA and treated with MG132 (10 µM for 16 h) and, where indicated, digested with trypsin. NP40 indicates the same microsomes-containing lysates treated with detergent to solubilise ER membranes, thus making also luminal proteins accessible to trypsin. (d) WB-ra of supernatants and cellular extracts from cells expressing HC-BAP (HC) and cyt-BirA with (+) or without (−) LC. All blots were developed with anti-SV5 mAb or anti-tubulin; the LC in (d), left panel, was visualized because of the secondary anti-mouse IgG antibody used.

We next performed experiments with two different secretory model proteins, NHK-α1AT and Ig HC, which led to essentially equivalent conclusions to those with MHC-Iα. In Fig. 5a the secretion-incompetent mutant NHK-α1AT was compared to the wild type protein. Compromised secretion of NHK-α1AT resulted in intracellular accumulation with a clear fraction of biotinylated molecules. In contrast, only a small fraction of intracellular α1AT was biotinylated, while the secreted material was, as expected, totally non-biotinylated. A smaller band of α1AT was also observed, and resulted to be biotinylated, likely representing a cytosolic fragment. In the presence of the proteasome inhibitor MG132 a band corresponding to de-glycosylated NHK-α1AT (confirmed by PNGaseF treatment, Fig. 5b) was detected, that was obviously fully biotinylated. However, as in the case of MHC-Iα, a fraction of glycosylated NHK-α1AT was also biotinylated. In addition, a smaller de-glycosylated band (because insensitive to PNGaseF) of mutant NHK-α1AT, which most likely corresponds to the same N-terminal deletion of α1AT shown in Fig. 5a, was fully biotinylated, consistent with cytosolic localization.

Also for NHK-α1AT the biotinylated fraction corresponded to cytosolically exposed molecules as shown in Fig. 5c. When microsomes-containing lysates prepared from cells co-expressing NHK-α1AT and cyt-BirA were treated with trypsin, the non-biotinylated material was trypsin-resistant, as it corresponds to ER protected molecules, while the whole fraction of biotinylated molecules was trypsin-sensitive.

In contrast, when microsome membranes were solubilized by detergent treatment (NP40), both biotinylated and non-biotinylated molecules became trypsin-sensitive. The biotinylated proteolytic fragments (containing SV5-BAP) generated by trypsin bound to StrAv and migrated as a smear roughly in the same position of NHK-α1AT (Fig. 5c, lanes 4, 6).

Expression of the second secretory model protein, Ig γHC, in the absence of LC resulted in no secretion and in the appearance of a significant amount of intracellular biotinylated molecules. In contrast, co-expression with LC promoted, as expected, active secretion of non-biotinylated HC and a very reduced level of the intracellular biotinylated fraction, confirming that the presence of LC rescues HC from ERAD, reducing the extent of retro-translocation (Fig. 5d).

### Retro-translocation of calreticulin

**Figure 6:**
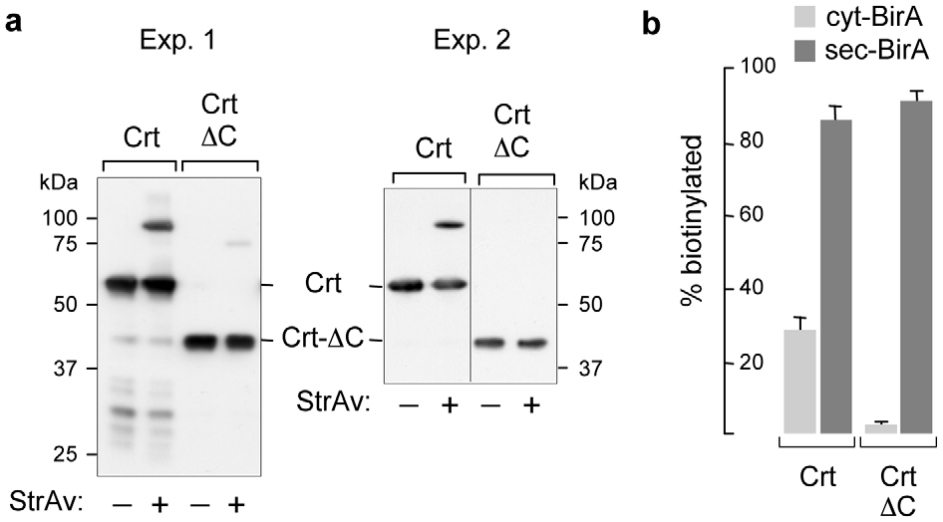
Retro-translocation of calreticulin. (a) WB-ra of cellular extracts (developed with anti-SV5 mAb) of HEK293 cells co-transfected with Crt or the mutant Crt-ΔC and cyt-BirA. Two representative different experiments are shown. (b) Quantification of the relative levels of biotinylation of Crt and Crt-ΔC (obtained from the WB-ra) when co-expressed with cyt-BirA or sec-BirA. Histograms show the results of three independent experiments; error bars indicate one standard deviation.

Finally, we determined the level of biotinylation associated to the spontaneous retro-translocation of Crt and compared to the dislocation-incompetent mutant (Crt-ΔC), which lacks the 115 residues long C-terminal portion. As shown in two different representative experiments in Fig. 6, up to 30% of Crt was biotinylated, in agreement with active dislocation activity and with the relative abundance of dislocated Crt reported [9]. In contrast, less than 5% dislocation was observed for Crt-ΔC. As expected, however, both of them were almost fully biotinylated by sec-BirA (Fig. 6b). In addition, a number of smaller fragments (representing deletions from the C-terminus since the tag was at the N-terminus) was sometimes detected (Fig 6a, experiment 1) for Crt, but not for the Crt-ΔC mutant. These fragments appeared not to be generated by cytosolic proteases, since they were not biotinylated when co-expressed with cyt-BirA, but were so with sec-BirA (not shown).

### Determination of retro-translocation by ELISA

**Figure 7:**
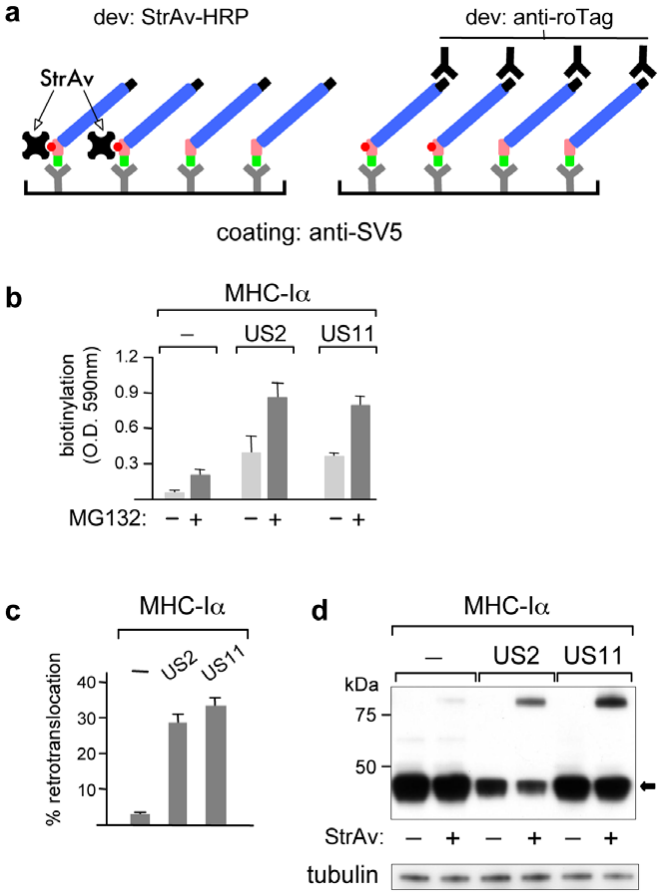
Determination of retro-translocation by ELISA. (a) Scheme of the ELISA used to monitor biotinylation of the retro-translocated fraction. Anti-SV5 mAb coating ensures capture of both, biotinylated and not-biotinylated BAP-MHC-Iα, which are then revealed with HRP-conjugated StrAv (only biotinylated MHC-Iα) and with anti-roTag (total MHC-Iα). (b) Retro-translocation levels (developed with HRP-conjugated StrAv) of BAP-MHC-Iα in HEK293 cells transfected alone or with US2 or US11, in the presence or absence of MG132 (50 µM for 4 h), as indicated. (c) Proportion of retro-translocated BAP-MHC-Iα (developed in parallel with HRP-conjugated StrAv and anti-roTag) in HEK293 cells transfected alone or with US2 or US11, expressed as the fraction of biotinylated BAP-MHC-Iα relative to the total amount of BAP-MHC-Iα. Histograms show the results of three independent experiments; error bars indicate one standard deviation. (d) WB-ra of samples used in (c), developed with anti-SV5 mAb or anti-tubulin. The arrow indicates the position of the non-biotinylated MHC-Iα.

Biotinylation also allows detection and quantification by ELISA of the level of retro-translocation as well as the proportion of dislocated molecules. We show it for MHC-Iα. The assay was set up by coating plates with anti-SV5 to capture all MHC-Iα molecules regardless of their state of biotinylation, and then revealed: i) with HRP-conjugated StrAv to determine the level of biotinylation, or ii) with HRP-conjugated StrAv (for the biotinylated fraction) and in parallel, with a second antibody (for the total amount of protein) to determine the proportion of retro-translocated molecules (Fig. 7a). In our case we used a BAP-MHC-Iα construct that contained, in addition to the SV5 at the N-terminus, a second tag (roTag) at the C-terminus (BAP-MHC-Iα-roTag). To normalize the assay, we used an extract of cells co-transfected with the same BAP-MHC-Iα-roTag construct and sec-BirA, since in that case all molecules are biotinylated. The extent of retro-translocation was therefore determined as the fraction of biotinylated molecules (revealed with StrAv) relative to the total amount of MHC-Iα in the sample (revealed with anti-roTag). Fig. 7b shows the levels of biotinylation of MHC-Iα expressed with US2 or US11 in the absence and presence of MG132, while Fig. 7c shows a plot of the percentage of dislocated MHC-Iα in cells co-expressing US2 or US11. The values in Fig. 7c are consistent with those obtained, for the same samples, with the WB-ra (Fig. 7d).

Taken together the data obtained with the different models demonstrated that biotinylation by cyt-BirA of proteins within the secretory pathway (properly BAP-tagged on the ER luminal side) is highly specific for molecules that have been retro-translocated from the ER to the cytosol, thus representing a fast and reliable way to determine the extent of ER-to-cytosol retro-translocation.

## Discussion

Biotinylation of proteins, either chemical or enzymatic, is a widely used technology with a variety of applications in biological research. Enzymatic biotinylation, however, offers the possibility to be implemented *in vivo* to achieve specific labeling of pre-defined proteins that have been properly tagged with the 15 aa-long biotin acceptor peptide BAP [22], which contains a single lysine as a biotin acceptor residue. The *in vivo* biotinylation in mammalian cells by the *E. coli* derived BirA is highly efficient and specific for the BAP peptide, resulting in a mono-biotinylated product. We have previously shown that cyt-BirA co-expressed with a cytosolically located protein (rotavirus NSP5) resulted in complete biotinylation of the target [23]. Similarly, the MHC-Iα-BAP, with the BAP tag localized to the cytosolic side was completely biotinylated by cyt-BirA. In addition, biotinylation is extremely stable: the only de-biotinylating activity in cells takes place on short peptides derived from degraded proteins and in particular by the activity of biotinidase, an enzyme that removes biotin from biocytin (Biotinyl-L-lysine [31]). In fact, we observed no decrease in the amount of biotinylated proteins present in cellular extracts following incubation of lysates for several hours. In our method we exploit all these characteristics of BirA in addition to its cytosolic localization, to specifically label molecules that undergo ER-to-cytosol retro-translocation.

Using the four different model-proteins selected, that represent well-known examples of dislocation, we showed that biotinylation identifies retro-translocated molecules for different types of proteins (membrane-bound, secretory and ER proteins). Taken together the data we presented clearly demonstrate that only molecules exposed to the cytosolic side become substrate of BirA and therefore biotinylated. Biotin labeling allows, either by the WB-ra or by ELISA, to precisely establish the fraction of biotinylated molecules, making therefore possible to determine the level of retro-translocation for a given protein in different conditions, as well as to compare the relative folding efficiency of different proteins or of the same protein in different cells. For instance, we have observed that, while in HEK293 and HeLa cells US2 induces a higher level of MHC-Iα degradation than US11, in CHO cells US11 performs significantly better than US2.

So far, just a few techniques have been described for the detection of retro-translocated molecules in the presence of proteasome inhibitors, to favor accumulation of retro-translocated molecules. For example, retro-translocation of MHC-Iα, and also of other proteins, is usually identified as the fraction of cytosolic de-glycosylated intermediates, which requires the use of proteasome inhibitors to accumulate [30], [20]. In contrast, our method was able to detect a significant amount of biotinylated molecules of MHC-Iα as well as secretory NHK-α1AT and HC that were still glycosylated, even in the absence of proteasome inhibition. As expected, because of their cytosolic localization the few de-glycosylated molecules detected in the presence of proteasome inhibitors were completely biotinylated.

Therefore, by *in vivo* biotinylation it is possible to study the rate and extent of retro-translocation also for non-glycosylated proteins and, in general, regardless of the glycosylation status. Moreover, biotinylation of BAP-tagged proteins can be adapted to *in vitro* retro-translocation assays.

We noticed that in HEK293 cells the accumulation of de-glycosylated MHC-Iα was less relevant than what described for U373 cells [32], as previously reported also for the non-classical class I molecule HFE in HEK293 cells [33]. *In vivo* biotinylation appears to be very efficient in labeling recently retro-translocated molecules, well before their de-glycosylation. De-glycosylated molecules are only detected following proteasome inhibition, thus suggesting that de-glycosylation is the rate-limiting step in the degradative pathway. The results obtained indicate that quantifying retro-translocation by considering only de-glycosylated molecules may result in underestimation of the extent of retro-translocation. On the other hand, treatment of cells with proteasome inhibitors can lead, as a consequence of accumulation of misfolded proteins, to the activation of the unfolded protein response (UPR), which implies increased expression of proteins involved in protein folding, such as chaperones, and proteins directly participating in retro-translocation, such as SEL1L and Derlin proteins [34], [35]. Consistently, in our experiments treatment with proteasome inhibitors led to an increase in the amount of retro-translocated/biotinylated MHC-Iα molecules accumulated even in the absence of immunoevasins.

Biotinylated molecules can be directly detected by assaying blotted membranes with StrAv. However, we prefer the retardation assay because it allows also detection of the non-biotinylated fraction and of the possible presence of fragments, which may not be biotin-labeled, as we have observed for Crt. In this respect, it is not clear what those Crt fragments represent. They were all derived from deletions of the C-terminus (since the tag is N-terminally located) with similar or shorter lengths than the Crt-ΔC mutant, which lacks 115 aa. It is possible that these fragments were generated and retained in the ER, and not retro-translocated. Alternatively, they may result from the activity of lysosomal proteases participating in degradation of ER material as a consequence of autophagy [36]. Interestingly, since Crt retro-translocation appears to be of relevance for its nuclear activity, it may not be related to the turnover of the protein via proteasomal degradation. It thus remains an open question whether natural turnover of Crt involves autophagy rather than proteasomal degradation.

A further advantage offered by the described technique is the possibility to affinity purify biotinylated molecules, making possible the analysis, for instance by mass-spectrometry, of intermediates of retro-translocation.

In summary, the mono-biotinylation of cytosolically dislocated molecules here described represents a novel, simple, quantitative and reliable way of determining the extent of ER-to-cytosol retro-translocation of proteins *in vivo*.

## Materials and Methods

### Ethics statement

An ethics approval for the use of human RNA was not requested, since the material used was already available from a previous study [37], and it was obtained at a time when there was no such requirement.

### Constructs

The human MHC-Iα allele A2 cDNA (accession number U02935) was PCR amplified from RNA extracted from anonymous healthy donors lymphocytes and inserted into pcDNA3 expression vectors (Invitrogen) containing the coding sequences for a secretion signal, the SV5 tag (GKPIPNPLLGLD) and the biotin acceptor peptide BAP (GLNDIFEAQKIEWHE [22]). Two plasmids were generated, one containing the SV5 and BAP sequences at the aminoterminal side of MHC-Iα (pcDNA-BAP-MHC-Iα) and one with BAP and SV5 sequences fused to the carboxyterminal side of MHC-Iα (pcDNA-MHC-Iα-BAP, Fig. 1b). The cDNA for the human α1-antitrypsin (α1AT, accession number K01396) was similarly PCR amplified and inserted in a vector that adds the SV5 and BAP sequences at the carboxyterminal end (pcDNA-α1AT-BAP, Fig. 1b). The vector expressing the NHK mutant of α1AT [38] was generated by substituting the wild type α1AT sequence in the pcDNA-α1AT-BAP vector with a sequence containing a two-base deletion after codon 318 of the mature protein resulting in the insertion of 14 frame-shifted codons (pcDNA-NHK-α1AT-BAP, Fig. 1b). The plasmid expressing the mouse γ heavy chain was obtained by inserting the coding sequence in the pcDNA vector with the SV5 and BAP sequences at the carboxyterminal end (pcDNA-HC-BAP, Fig. 1b). The vectors expressing the full-length and truncated calreticulins were generated by inserting the PCR amplified cDNAs of human calreticulin (accession number NM_004343) or the calreticulin truncated after codon 285 of the mature protein in the vector with the SV5 and BAP sequences at the aminoterminus (pcDNA-BAP-Crt and pcDNA-BAP-Crt-ΔC, Fig. 1b).

### Cell culture and transfection

HEK293 cells were grown in Dulbecco's modified Eagle's medium (DMEM), supplemented with 10% fetal calf serum (FCS).

Cells were co-transfected in 6-well plates (about 5×10^5^ cells/well) by standard calcium phosphate technique [39]. When indicated, US2 or US11 plasmids (kindly provided by D. Tortorella) were co-transfected. In transfection experiments involving mouse γHC, a vector expressing a mouse κLC was co-transfected, where required. 18 h after transfection, medium was discarded and replaced by 2 ml of serum free medium supplemented with 0.1 mM biotin and further incubated for at least 8 h. When required, after 4 h incubation with biotin, the proteasome inhibitors MG132 (Sigma) or Bortezomib (Selleck Chemicals) were added at a concentration of 50 µM for 4 h, or at 10 µM for 16 h (MG132).

### Cell extract preparation, gel retardation assay and Western blotting

HEK293 transfected cells were lysed directly in the transfection plates, after collecting medium and washing with PBS to remove free biotin, with 100 µl/well of SDS-lysis buffer (100 mM Tris-HCl, pH 6.8, 6% SDS) and subsequently sonicated to disrupt nuclear DNA. For gel retardation assay, samples denatured in SDS-gel-loading buffer (25 mM Tris-HCl, pH 6.8, 1% SDS, 10% glycerol, 175 mM β-mercaptoethanol) were boiled for 10 min, cooled to RT and incubated with 1 µg of StrAv (Sigma) for 30 min before separation on SDS-PAGE, then transferred to PVDF membranes for immunodetection with anti-SV5 mAb followed by incubation with HRP-labeled anti-mouse whole IgG (Jackson) and ECL reaction. Quantification of bands was performed with the image processing software Image-J 1.43u, National Institutes of Health, USA. Purification of biotinylated MHC-Iα was carried out by incubating the lysates in SDS-PAGE loading buffer with StrAv-coated magnetic beads (Dynabeads, Invitrogen) and eluting by boiling for 10 min. Where indicated, eluted material was treated with Peptide-N-Glycosidase-F (PNGase-F, New England Biolabs) according to manufacturer indications.

### Cytofluorimetric analysis

Cells were, 24 h after transfection, incubated either with the anti-SV5 mAb followed by fluorescein-conjugated anti-mouse IgG (KPL), or with QuantumDot_655_-Streptavidin (Invitrogen) and analyzed in a FACSCalibur (Becton Dickinson).

### Trypsin sensitivity assay

Microsome-containing lysates were obtained by resuspending cells in buffer 50 mM Tris-HCl pH 8.0, 250 mM sucrose and 10 mM N-ethyl-maleimide (to block cyt-BirA post-lysis activity), freezed and thawed once, and centrifuged at 5000×g for 5 min at 4°C. Supernatants (microsome-containing lysates) were incubated with 1 µg trypsin for 1 h at 37°C. When indicated NP40 was added at 0.5% final concentration.

### Quantification of retro-translocation by ELISA

Samples in SDS-lysis buffer were treated with 0.15 M β-mercaptoethanol and boiled for 10 min. Free β-mercaptoethanol was then quenched by diluting with an equal volume of iodoacetamide 0.15 M in buffer Tris-HCl 0.1 M, pH 8.0. Serial dilutions of these lysates in buffer TNN (50 mM Tris-HCl, pH 8.0, 250 mM NaCl, 0.5% NP-40), starting from 10 µl/well, were applied to polystyrene microplates (Nunc Maxisorp C96) coated with 0.2 µg/ml of anti-SV5 mAb in buffer NaHCO_3_-Na_2_CO_3_ 50 mM, pH 9.5 (100 µl/well). Proteins were bound to the plates for 1 h at RT and then reacted with either HRP-labeled StrAv (Jackson Immunoresearch) or anti-roTag (scFv in SIPε_S2_ format [40]) followed by HRP-labeled anti-human IgE (KPL), and developed with TMB reagent (Sigma). After blocking with H_2_SO_4_, the O.D. at 450 nm was read on a BioRad microplate reader 550. To calculate the proportion of retro-translocated molecules, the ratio between the slopes derived from the linear region of the serial dilutions curves developed with StrAv and anti-roTag was first determined for the 100% biotinylated sample (MHC-Iα co-expressed with sec-BirA), and termed reference factor, F_100_. The same factor was then determined for each sample (F_x_). The biotinylated fraction was thus calculated as (F_x_/F_100_)×100.

## Supporting Information

Figure S1 **Pulse-chase labeling of retro-translocated MHC-Iα.** PAGE retardation assay of anti-SV5 immunoprecipitated cellular extracts of HEK293 cells co-transfected with BAP-MHC-Iα and cyt-BirA and, where indicated, with US2. Cells were starved for 30 min with Methionine/Cysteine free medium, supplemented with 10% of dialyzed FCS and 0.1 mM biotin, then labelled for 30 min with 200 µCi/ml [^35^S]-Methionine/Cysteine (Perkin Elmer) and chased in biotin-containing fresh medium for 120 min. Cells were then lysed in 100 µl of SDS-lysis buffer, diluted with 400 µl of TNN and digested with DNaseI for 1 h to disrupt DNA. Samples were immunoprecipitated with anti-SV5 mAb and Protein A-agarose (Repligen) and resolved in a 10% SDS-PAGE. The arrow indicates the position of the non-biotinylated MHC-Iα. Right panel, quantification of the BAP-MHC-Iα biotinylated band, expressed as percentage of the total immunoprecipitated BAP-MHC-Iα (biotinylated+non-biotinylated). Histograms show the results of three independent experiments; error bars indicate one standard deviation.
(TIF)
