# Supplementary figures and images for: Efficient Detection of Proteins Retro-Translocated from the ER to the Cytosol by In Vivo Biotinylation
Source: PLoS One. 2011 Aug 24;6(8):e23712. doi: 10.1371/journal.pone.0023712 (PMC3161056; doi:10.1371/journal.pone.0023712)

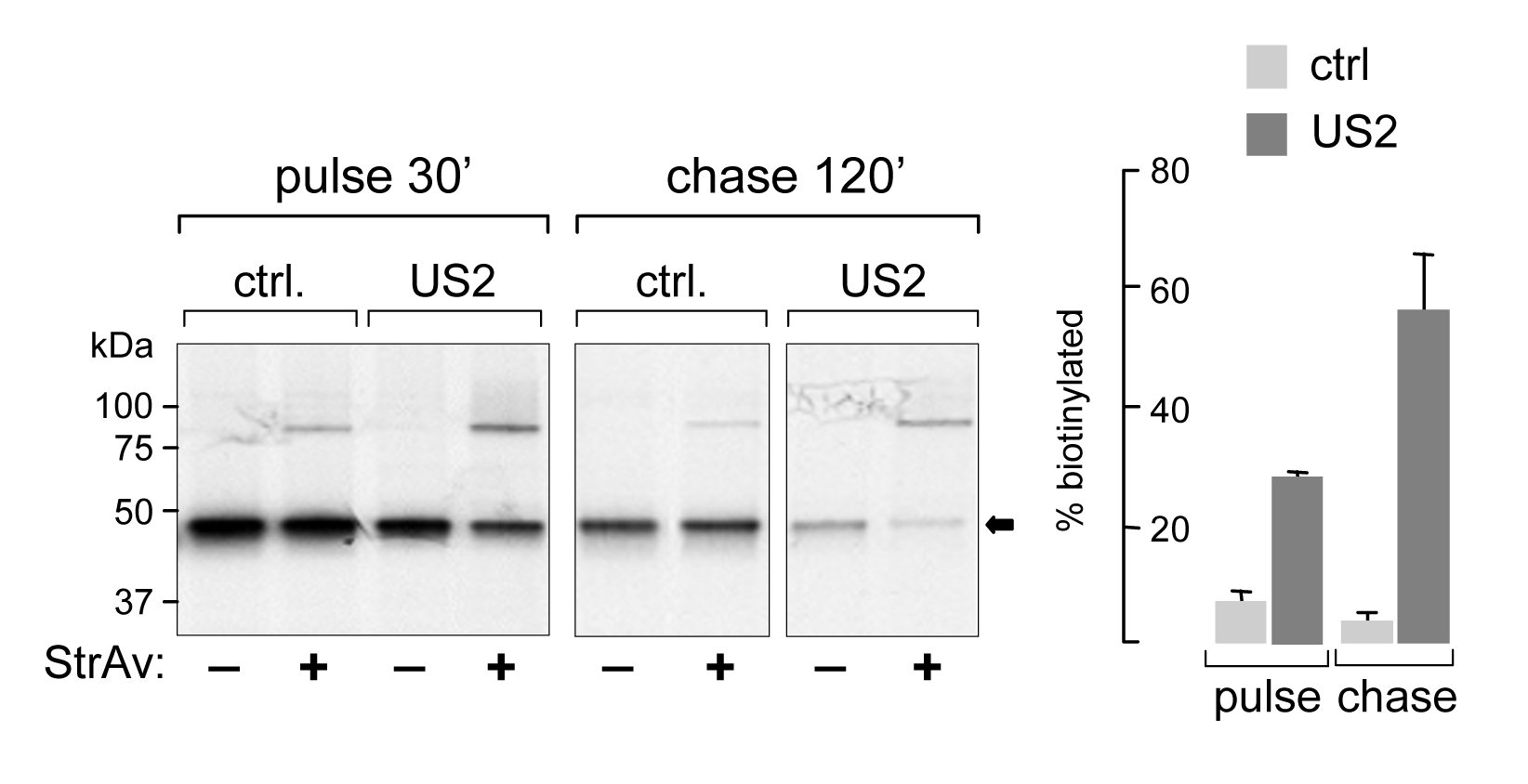

Supplement: Figure S1 — Pulse-chase labeling of retro-translocated MHC-Iα. PAGE retardation assay of anti-SV5 immunoprecipitated cellular extracts of HEK293 cells co-transfected with BAP-MHC-Iα and cyt-BirA and, where indicated, with US2. Cells were starved for 30 min with Methionine/Cysteine free medium, supplemented with 10% of dialyzed FCS and 0.1 mM biotin, then labelled for 30 min with 200 µCi/ml [35S]-Methionine/Cysteine (Perkin Elmer) and chased in biotin-containing fresh medium for 120 min. Cells were then lysed in 100 µl of SDS-lysis buffer, diluted with 400 µl of TNN and digested with DNaseI for 1 h to disrupt DNA. Samples were immunoprecipitated with anti-SV5 mAb and Protein A-agarose (Repligen) and resolved in a 10% SDS-PAGE. The arrow indicates the position of the non-biotinylated MHC-Iα. Right panel, quantification of the BAP-MHC-Iα biotinylated band, expressed as percentage of the total immunoprecipitated BAP-MHC-Iα (biotinylated+non-biotinylated). Histograms show the results of three independent experiments; error bars indicate one standard deviation. (TIF) [file pone.0023712.s001.tif]
